# Evaluation of deep space exploration risks and mitigations against radiation and microgravity

**DOI:** 10.3389/fnume.2023.1225034

**Published:** 2023-09-21

**Authors:** William Dobney, Louise Mols, Dhruti Mistry, Kevin Tabury, Bjorn Baselet, Sarah Baatout

**Affiliations:** ^1^ Radiobiology Unit, Belgian Nuclear Research Centre (SCK CEN), Mol, Belgium; ^2^ School of Aeronautical, Automotive, Chemical and Materials Engineering, Loughborough University, Loughborough, United Kingdom; ^3^ Department of Physics and Astronomy, KU Leuven, Leuven, Belgium; ^4^ Department of Biomedical Engineering, College of Engineering and Computing, University of South Carolina, Columbia, SC, United States; ^5^ Department of Molecular Biotechnology, UGhent, Gent, Belgium; ^6^ Department of Human Structure & Repair, UGhent, Gent, Belgium

**Keywords:** space radiation, microgravity, risk assessments, mitigations, radiation protection

## Abstract

Ionizing radiation and microgravity are two considerable health risks encountered during deep space exploration. Both have deleterious effects on the human body. On one hand, weightlessness is known to induce a weakening of the immune system, delayed wound healing and musculoskeletal, cardiovascular, and sensorimotor deconditioning. On the other hand, radiation exposure can lead to long-term health effects such as cancer and cataracts as well as have an adverse effect on the central nervous and cardiovascular systems. Ionizing radiation originates from three main sources in space: galactic cosmic radiation, solar particle events and solar winds. Furthermore, inside the spacecraft and inside certain space habitats on Lunar and Martian surfaces, the crew is exposed to intravehicular radiation, which arises from nuclear reactions between space radiation and matter. Besides the approaches already in use, such as radiation shielding materials (such as aluminium, water or polyethylene), alternative shielding materials (including boron nanotubes, complex hybrids, composite hybrid materials, and regolith) and active shielding (using fields to deflect radiation particles) are being investigated for their abilities to mitigate the effects of ionizing radiation. From a biological point of view, it can be predicted that exposure to ionizing radiation during missions beyond Low Earth Orbit (LEO) will affect the human body in undesirable ways, e.g., increasing the risks of cataracts, cardiovascular and central nervous system diseases, carcinogenesis, as well as accelerated ageing. Therefore, it is necessary to assess the risks related to deep space exploration and to develop mitigation strategies to reduce these risks to a tolerable level. By using biomarkers for radiation sensitivity, space agencies are developing extensive personalised medical examination programmes to determine an astronaut's vulnerability to radiation. Moreover, researchers are developing pharmacological solutions (e.g., radioprotectors and radiomitigators) to proactively or reactively protect astronauts during deep space exploration. Finally, research is necessary to develop more effective countermeasures for use in future human space missions, which can also lead to improvements to medical care on Earth. This review will discuss the risks space travel beyond LEO poses to astronauts, methods to monitor astronauts' health, and possible approaches to mitigate these risks.

## 1. Introduction

With the current desire of space-faring nations to travel back to the Moon, the world will soon turn its attention to Mars once a lunar base has been established. However, with a mission profile unlike anything astronauts have flown before, such a mission will be extremely challenging. It involves traveling 50 million kilometres to reach Mars. The distance between the planets is so large that there will be latency of up to 20 min in voice and data transmissions between mission control on Earth and a base on Mars. As a result, neither the surface habitat nor the systems on board the transit spacecraft will be under the real-time control of the ground support team. The onboard inventory of equipment and supplies needs to be strategically arranged in advance because cargo resupply from Earth will not be possible. The size, quantity, and functionality of onboard equipment will also be restricted by volume, mass, and power parameters. All vehicle systems must be powerful and reliable because there will be less ground supervision. The systems and astronauts must also work independently of ground support. In order to handle the inevitable critical crises that may occur, astronauts will be dependent on their own capabilities and onboard supplies.

Today, we have numerous technological obstacles that prevent us from sending a human expedition to Mars. We will need to devise procedures for utilizing *in situ* resources before launching the first crew. Future astronauts will need to create some of these consumables from local space-based resources instead of bringing enormous quantities of oxygen, water, and propellant with them from Earth (1). We will require systems to land heavier payloads (up to 40 tonnes of equipment and supplies for a human trip) on planetary surfaces, and we will need ion propulsion systems to shorten journey durations to interplanetary destinations. Before humanity travels into outer space, these and other advancements will be required.

Maintaining the health of the astronauts is considered to be one of the biggest barriers (2) for deep space exploration. It will no longer be possible for ground-based medical professionals to monitor astronaut health as they have in the past, especially in an emergency. A deep space mission cannot be aborted in order to return an injured or unwell crew member to Earth for treatment. Future crews need to be fully trained and capable of managing their own health. Imaging, surgery, emergency treatment, and laboratory examinations of blood, urine, and other biospecimens must all be available as onboard medical resources. There should be at least one physician on the crew who has experience practicing remote medicine.

The environment in outer space is dangerous. As ionizing radiation and microgravity/weightlessness are the two main risks going into space, it is of importance to review the consequences and possible health effects and ways of mitigating those space stresses (3). However, little data are currently available on radiation and microgravity effects. The data gathered pertaining to space radiation are obtained from atomic bomb survivors or those exposed to chronic radiation for medical purposes, whereas the data pertaining to microgravity exposure are based on a combination of data obtained from prior space missions and experiments conducted in simulated microgravity (4, 5).

This article will first focus on a detailed definition and description of cosmic radiation and microgravity. Thereafter, the effects of these risks on health are described. Lastly, several types of protection and countermeasures are reviewed and discussed.

## 2. Ionizing radiation

Ionizing radiation is one of the main dangers astronauts face during their missions. Before traveling into deep space, numerous concerns related to radiation must be resolved. Can radiation protection strategies already employed in LEO be modified for use in outer space? Can low atomic weight materials be used in the design of spacecraft destined for deep space in order to protect crews? Could a safe haven compartment offer the crew protection against severe radiation exposure during a significant solar particle event whilst in transit to a planet? Could Martian regolith serve as a lining for underground habitats to provide protection? Will shielding be enough to reduce exposure on its own, or will biological and pharmaceutical countermeasures also be required? This section intends to describe the different types of space radiation and their effects on astronauts.

### 2.1. Definition and description

**Figure 1:**
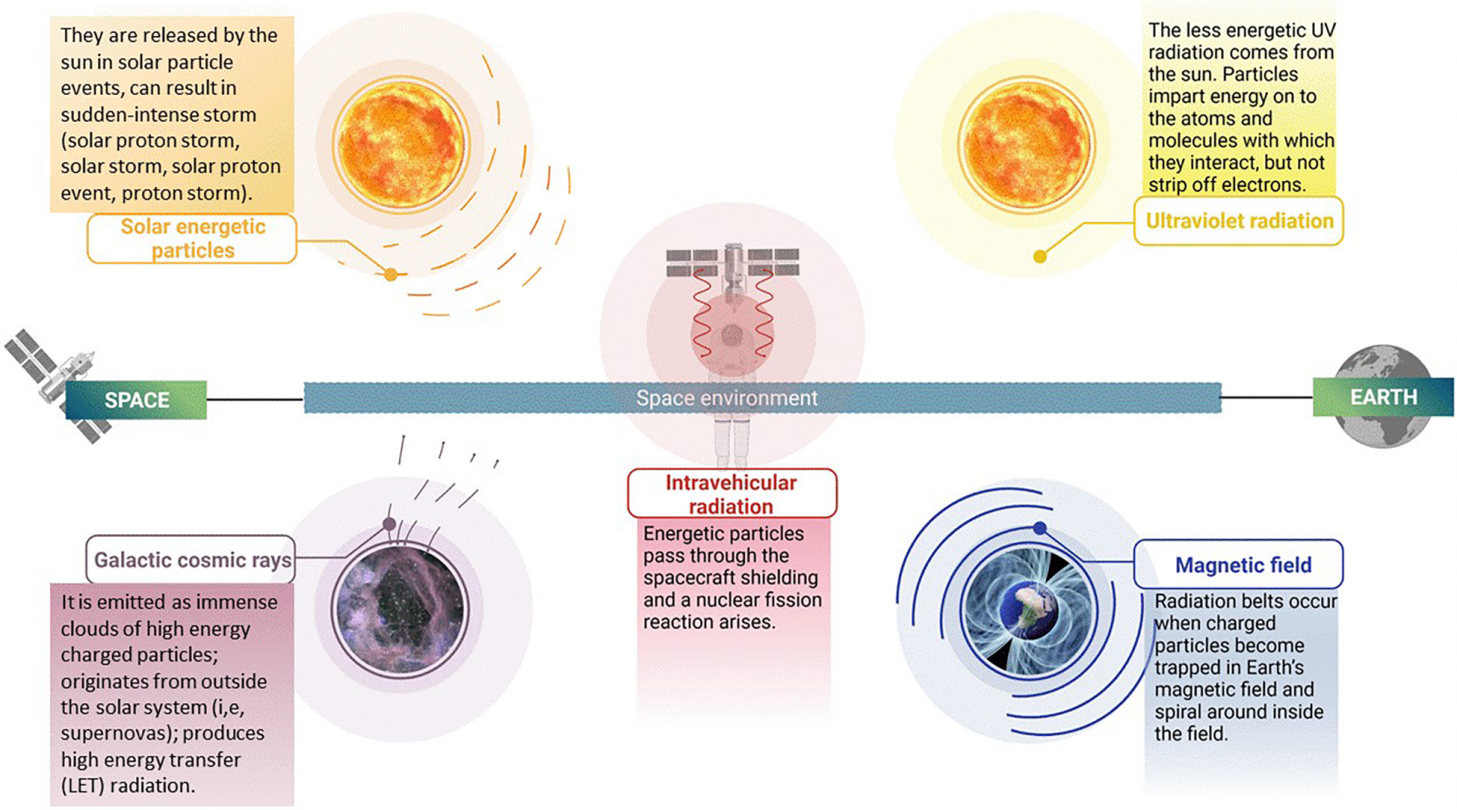
Types of ionizing radiation in space: illustration depicting the various types of ionizing radiation present in the space environment. These include solar energetic particles, galactic cosmic rays, ultraviolet radiation, intravehicular radiation and magnetic field. The figure highlights the distinct characteristics of each type of radiation, such as their composition and energy levels, which contribute to their unique effects on biological systems and materials. Created with BioRender.com.

Radiation is a form of energy that is emitted or transmitted in the form of electromagnetic waves and/or energised particles (6, 7). There are two kinds of radiation, ionizing and non-ionizing radiation. Ionizing radiation has particles that have enough energy to remove electrons from (ionise) atoms or molecules. Non-ionizing radiation may also be dangerous; however, it can be more easily shielded, e.g., by using sunglasses (6, 7). Missions going beyond Earth's magnetosphere will encounter different forms of ionizing radiation. Solar winds or flares are the main source of energetic particles, which are emitted constantly by the Sun. Levels of these solar flares will vary depending on the 11-year cycle of sun activity. These solar particle events (SPE) produce large plasma clouds, which carry highly energetic protons and heavy ions. Moreover, Galactic Cosmic Radiation (GCR), with its origin being from different solar systems, is another type of radiation. GCR contains high energetic ions that move at relativistic speeds (8). Exposure to both GCR and SPE could have a major effect on the health of astronaut crews (3). Different types of space radiations are shown in Figure 1.

#### 2.1.1. Galactic cosmic radiation

GCR ions, originating from outside our solar system, produce high linear energy transfer (LET) radiation. GCR is composed primarily of protons (87%), helium (12%) and heavier nuclei (1%) (9–11). Those particles that come into contact with biological tissue or other (in)organic materials have sufficient energy to penetrate several centimetres of material (12). When transiting outside of LEO, it has been estimated that a hydrogen ion will pass through every cell nucleus of an astronaut crew member every day, and heavier High (H) atomic number (Z) and high energy (E) (HZE) nuclei every few months (13). These HZE ions pose an enormous threat to human health because of their very high LET values. Shielding is currently used to reduce the radiation penetrating the spacecraft, but this means of protection is limited by the practical capabilities of current launch systems (3).

#### 2.1.2. Solar particle events

Solar energetic particles, which are emitted by solar flares and Coronal Mass Ejections (14), produce large quantities of energetic protons (9–11). Their energies are proportional to the sun's peak activity when equatorial sunspot activity is at its largest stage. The phase (11-year cycle) of the sun during an SPE has no effect on its intensity. Furthermore, the largest measured SPEs have occurred during off-peak periods of the 11-year cycle (3). Therefore, spacecraft designers must still consider this issue and ensure that these energies are shielded by the spacecrafts' hulls during LEO missions. Nevertheless, there is a possibility that astronauts are exposed to this form of radiation during extravehicular activities (EVA) and in poorly shielded environments (13).

#### 2.1.3. Intravehicular radiation

The health of astronauts is affected not only by SPE and GCR particles, but also by the interaction of these energetic particles with the spacecraft structure. When these particles pass through the spacecraft shielding, a nuclear fission reaction occurs in most cases, generating a plethora of secondary radiation composed of beta particles, x-rays, gamma rays, neutrons, protons, alpha particles, and heavy-charged particles (3).

### 2.2. Effects of radiation on health

**Figure 2:**
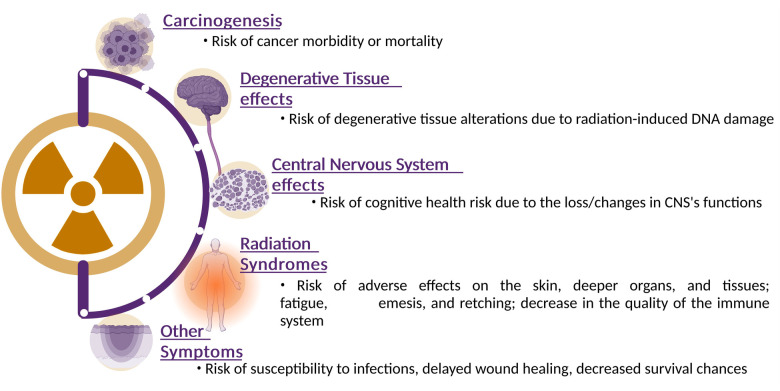
Health effects of space radiation: this illustrative figure offers a comprehensive visualization of the intricate and multifaceted health effects induced by space radiation exposure. The diagram indicates the major physiological risks that can occur in response to ionizing radiation encountered in space environments. Ionizing radiation can cause single-strand breaks, double-strand breaks, and oxidative damage to DNA. Starting from the top, the figure emphasizes the potential for genetic mutations and disruptions to DNA repair mechanisms, contributing to long-term health risks such as cancer. Transitioning to the tissue and organ level, the figure illustrates how cellular damage and responses can lead to tissue dysfunction. Emphasis is placed the potential for acute effects, such as radiation syndromes, as well as chronic effects such as degenerative tissue effects and impaired organ function. Finally, the figure highlights the link between these immediate and systemic effects and their long-term health consequences. The illustration emphasizes the increased risk of cancer, cardiovascular diseases, neurodegenerative disorders, and other chronic conditions that astronauts might face over their lifetime. Created with BioRender.com.

Ionizing radiation will be present in higher fluences beyond the Earth's protective magnetosphere. Astronauts who are exposed to galactic cosmic radiation for an extended period may develop cataracts as well as delayed and impaired wound healing and degenerative tissue disorders. Cancer could also develop with a higher incidence at a later stage in life, and genetic alterations are another possibility. Finally, a significant SPE could cause acute radiation sickness, possibly death. The risks encountered by astronauts exposed to cosmic radiation are outlined in Figure 2 and are discussed below (15, 16).

#### 2.2.1. Degenerative tissue effects and carcinogenesis

The high-LET radiation of GCR particles can damage biomolecules, organelles, and other cellular structures. Studies have shown that increased exposure to ionizing radiation altered tissues in a manner similar to aging (3). In contrast to high-LET radiation, studies have demonstrated a relationship between high doses of low-LET radiation and heart diseases, cataracts, and premature aging, amongst others (17, 18). However, the results of studies on low doses of low-LET radiation have not always been consistent. At the basis of these degenerative diseases lies the induction of DNA damage. When a DNA molecule is damaged by ionizing radiation, e.g., by losing a base or base modification such as oxidation, cells will try to repair it by using repair systems in order to maintain integrity and functionality. When DNA replication occurs before repair ends, or when repair was unsuccessful at restoring the DNA, this can result in apoptosis, a form of programmed cell death, but also in mutations that can possibly lead to genome instability and ultimately carcinogenesis (19, 20). Studies relating higher doses of radiation in space to carcinogenesis are still in their infancy. The majority of these studies used animals, cell models or atomic bomb survivors as their sample population to investigate the effects of high doses of radiation. However, the effects of space radiation on carcinogenesis remain unclear due to the small sample size of astronauts in space and wide range of time and dose intervals (21).

#### 2.2.2. Central nervous system effects

Radiation can interfere with cognitive functions (22–25). Studies at the Space Radiation Laboratory at NASA demonstrated cognitive health risks during Beyond Low Earth Orbit (BLEO) missions. Other research has demonstrated that low doses of GCR radiation can affect learning and memory, and in drastic cases can even kill neuronal cells (26). Therefore, executive functions in astronauts may deteriorate over time (27).

#### 2.2.3. Radiation syndromes

A study by Kennedy et al. (28) investigated the effects of high doses of SPE on blood cells, immune system parameters and skin, using different animal models (29, 30). The damage to the DNA of larger species was irreversible and adverse effects on the skin, deeper organs, and tissues were observed. Furthermore, lower doses of SPE particles also induced a decline in the immune system's performance. Fatigue, emesis, and retching are some well-known consequences of exposure to high doses of radiation (3).

#### 2.2.4. Other symptoms

Due to intravehicular radiation, susceptibility to infections is also increased, together with delayed wound healing and even a decreased survival chance. However, studies revealed that the susceptibility to diseases after exposure to radiation depends on the individual. Hence, further research needs to take this inter-individual variation into account and is discussed later in this paper (31, 32).

## 3. Microgravity

In space, astronauts are subjected to a variety of gravity conditions that have an impact on fundamental biological functions. Most human spaceflights have taken place in LEO, which is between 160 and 2,000 kilometres above the surface of the planet. This region is still shielded from charged particle space radiation (33). On Earth, gravity pulls objects towards the centre of the planet. This is known as normal terrestrial gravity (1 g). Earth's gravity's pulling nature is resisted by the Earth's surface, and it is this combination of force that determines the structure of our musculoskeletal system and how it supports our body. Therefore, contact forces that provide a variety of mechanical stimulation that is necessary for the operation of numerous physiological systems are constantly applied to organisms on Earth (33).

The effects of physical exercise stresses on the weight-bearing skeleton are a clear example of how these mechanical contact forces affect the human body. For the body to be able to resist these greater stresses, an increased load during weight-bearing exercise induces increased musculoskeletal growth.

Furthermore, the Earth's gravitational pull is also felt by spacecraft near to Earth. This enables spacecraft to orbit around the planet. Astronauts and the spacecraft's cargo are in a state of free fall while in LEO orbit around the Earth (33), and experience microgravity with a gravitational force of about 1 × 10^–6^ g. Many of the physiological problems that astronauts experience in space, such as motion sickness and otolith dysfunction as well as cardiovascular, bone, and muscle deterioration, are caused by the absence of normal terrestrial gravity (1 g) and the ensuing lack of mechanical stimulation of cells and tissues. The environment of spaceflight during astronaut missions has revealed numerous rapid tissue degenerative effects. These include bone and muscle loss, cardiovascular deconditioning, delayed wound healing and bone fracture healing, and impaired immune function (34).

**Figure 3:**
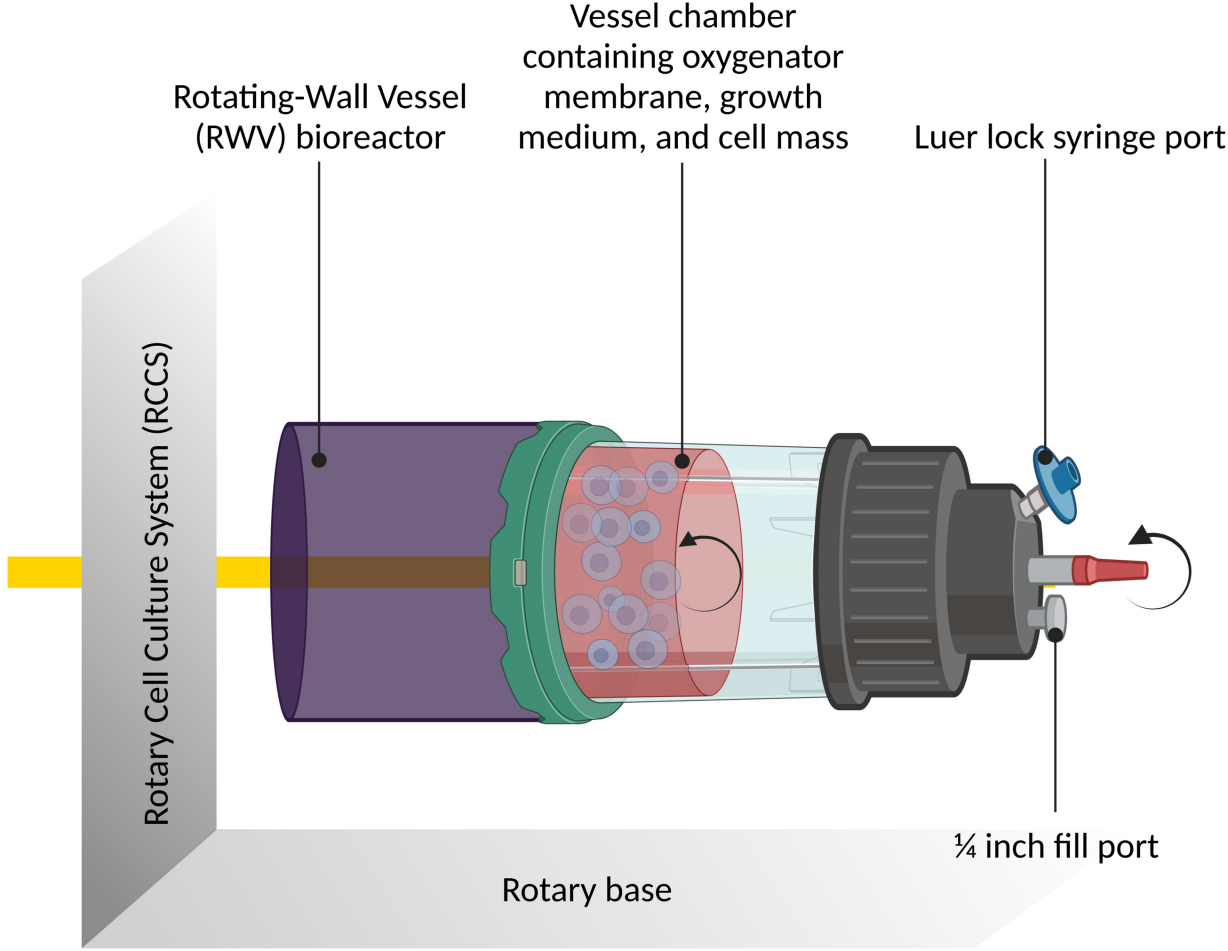
The rotary cell culture system containing the rotating-wall vessel bioreactor: schematic diagram illustrating the rotary cell culture system incorporating the rotating-wall vessel (RWV) bioreactor. The figure provides an overview of the bioreactor's design and its role in simulating microgravity conditions. It showcases how the RWV facilitates three-dimensional cell growth and enables studies on cellular responses under simulated weightlessness. Created with BioRender.com.

It is quasi-impossible, on Earth, to study effects in the absence of gravity, also referred to as weightlessness. On Earth, therefore, simulation models are used to replicate microgravity. By using such simulation models, it is possible to investigate the effects of microgravity on different cellular systems. The primary device used to simulate microgravity is the rotating-wall vessel also known as the slow rotating clinostat or rotating wall bioreactor (RWV; Figure 3).

In a controlled setting where gravitational forces are either reduced to a minimum or completely eliminated by continuous rotation, this laboratory apparatus is intended to imitate microgravity conditions for biological experiments. The apparatus consists of a cylindrical container that accommodates biological materials or cell cultures. An oxygen- and nutrient-rich growth medium that is inside the vessel provides the cells with what they require to live and develop. The rotating wall vessel's essential characteristic is its steady, continuous revolution. The biological samples inside the vessel undergo a constant shift in gravitational orientation as the RWV rotates. The samples enter a state of “free fall” as a result of this rotation, simulating near-weightlessness as the force of gravity is evenly dispersed throughout the cells (35–37). Another device is the Random Positioning Machine, which rotates biological samples along two distinct axes in a random pattern that removes any particular orientation (of the samples) in space (38). On Earth, this can be used to counteract gravity (39).

The revolving wall vessel is used by researchers to examine a variety of biological processes, including gene expression, immunological response, cell signaling, tissue development, and cell proliferation. It aids in the understanding of how microgravity affects cellular function and has possible implications for space travel, medicine, and regenerative medicine. It also sheds light on how organisms adapt to space conditions.

### 3.1. Effects of microgravity on health

**Figure 4:**
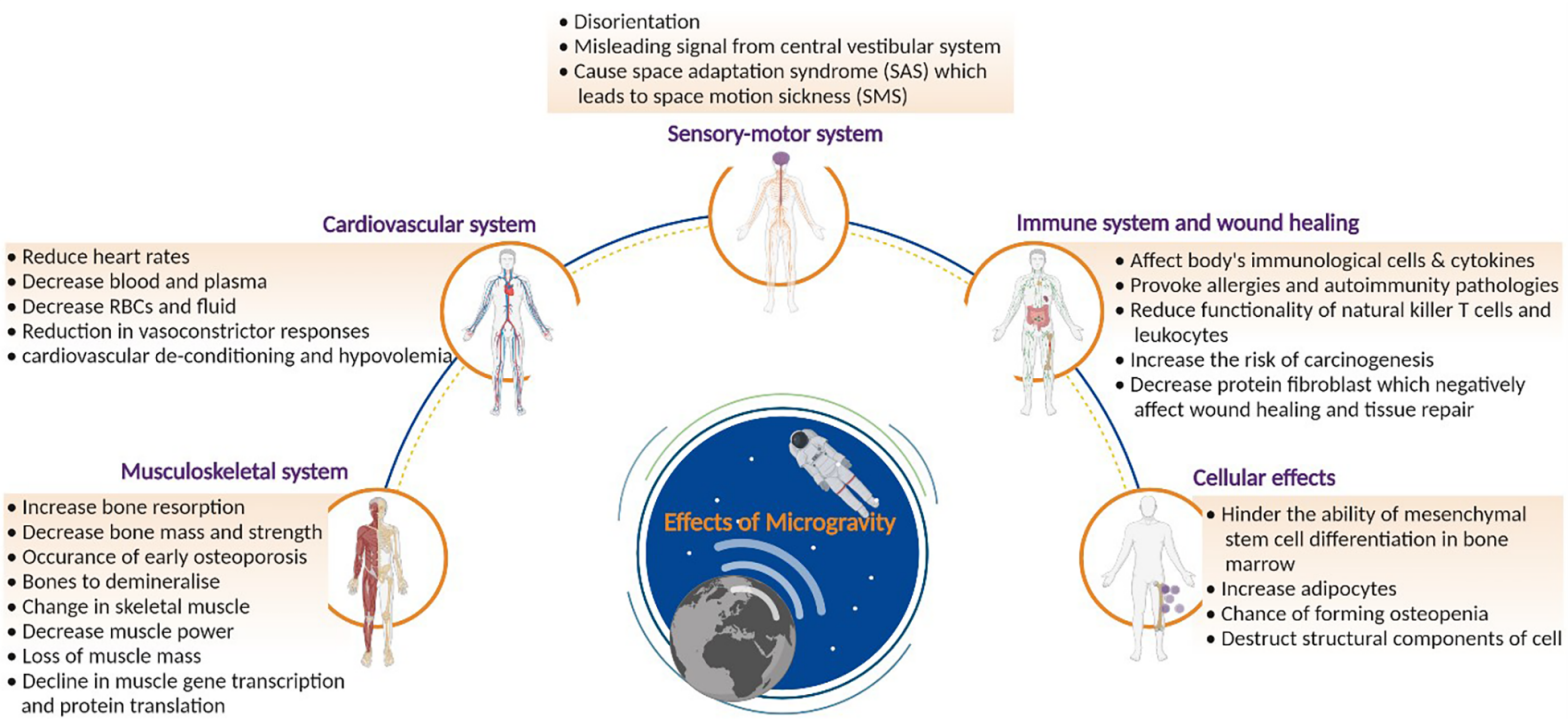
Effects of microgravity on health: comprehensive overview of the effects of microgravity on human health. The figure delves into physiological changes. By visualizing the impact on different bodily systems, the figure highlights the challenges posed by prolonged space missions on astronauts’ well-being. Created with BioRender.com.

A lack of gravity can have negative effects on human physiology, which are listed in Figure 4. The impact of microgravity can be observed on the musculoskeletal, cardiovascular, sensory-motor, and immune systems as well as on a cellular level. The following section details the impact on these systems.

#### 3.1.1. The musculoskeletal system

Studies have shown that microgravity can change bone tissue (40). This alteration in bone tissue can lead to a decrease in bone mass, an increase in bone resorption, and, accelerate osteoporosis (41–45). The function of the skeleton is three-fold: delivering mechanical integrity for movements and protection as well as controlling mineral homeostasis for metabolic paths (46). Under normal terrestrial conditions, mechanical stresses are exerted during bone formation. These stresses do not exist in microgravity, and their absence results in uncoupling of the bone formation and resorption mechanisms, leading to an increase in bone resorption (47, 48).

Changes in the muscle tissue, such as muscle atrophy, alterations in protein metabolism and stiffness, have also been observed (49, 50–53). The effects on antigravity muscles were deemed the most profound, as these muscles play a crucial role under standard terrestrial conditions. For example, the quadriceps help the body to remain upright. Moreover, amongst these antigravity muscles, the extensor muscles show a greater impact due to microgravity than the flexors. However, when considering long duration flights in space, all skeletal muscles are affected. Kalb & Solomon categorized the factors that cause a decrease in muscle functions into three groups (54). Firstly, the removal of antigravity load in the antigravity muscles leads to a reduction in muscle gene transcription and protein translation. The second factor is a decrease in neural drive. Microgravity changes the control and coordination resulting in a decreased muscle power. Thirdly, systematic factors such as hormone alterations and changes in metabolism contribute to a loss of muscle mass. Finally, upon returning to Earth, astronauts experience a “reloading process”. They encounter weakness and delayed onset muscle soreness due to the returning gravity, which results in muscle damage. This could be problematic when performing interplanetary missions (47). Therefore, astronauts must exercise in space to ensure muscle integrity.

#### 3.1.2. The cardiovascular system

Research has indicated a reduction in astronauts' heart rates when in space, related to the lower vascular resistance in space. Furthermore, blood and plasma volumes also change during spaceflight. Facial oedema and thinning of legs are observed because of the redistribution of some body fluids to the head. Moreover, plasma and blood volumes are decreased. This can be traced back to several factors. Firstly, a depressed urge to drink reduces fluid intake, leading to a reduction in urinary output. The diminished blood volume experienced in space arises from three factors. Firstly, a negative equilibrium caused by a decreased fluid intake coupled with a comparatively modest reduction in urine output. Secondly, fluid redistributions from the intravascular to the interstitial space are a consequence of diminished transmural pressure. The reduced transmural pressure is caused by a decrease in compression of all tissues, notably the thorax cage, from the reduced gravitational forces. Lastly, fluid transfers from the intravascular to the muscle interstitial space occur due to reduced muscular tension required for sustaining body posture (55). When astronauts return to Earth, post-flight orthostatic intolerance often occurs. Orthostatic intolerances are signs of the body's inability to compensate for the fast translocation of blood from the upper body to the lower body. Reduction in vasoconstrictor responses and other alterations during spaceflights, such as cardiovascular de-conditioning and hypovolemia (low circulation of blood volumes), can also be the cause of this intolerance. It is expected that similar disorders will occur during interplanetary missions. However, no studies exist that show whether reduced gravitational fields, such as that on Mars, could worsen or ameliorate the effects observed in microgravity. Further research is needed to guarantee the safety of astronauts during interplanetary missions (47).

#### 3.1.3. The sensory-motor system

Primarily due to microgravity, the anatomy of the human brain is known to change during spaceflight, according to several studies (56–60). Spaceflight causes the brain to shift upward within the skull (61, 62). Grey matter volume increases in the top of the brain and reduces near the base of the brain as a result (57, 63).

The displacement of cerebral fluid, including extracellular free water such as cerebrospinal fluid, occurs in conjunction with post-flight grey matter shifts. Following a spaceflight, there is an increase in cerebral fluid volume near the base of the brain and a decrease in cerebral fluid volume towards the top of the brain. Additionally, during spaceflight, the ventricles expand, with reported average volume increases ranging from 11% to 25% (56–60).

However, the spaceflight experience of current and past astronauts varies greatly. The normal duration of a space mission is between 2 weeks and 1 year, and there are both experienced and inexperienced astronauts (with various numbers of past flights and inter-mission gaps). It is not well understood if or how these individual variations in past flying experiences are related to the structural brain changes and intracranial fluid shifts brought on by spaceflight.

When people are exposed to a new gravity environment, their brains centrally reinterpret information from various sensory sources to create a sensorimotor state that is suitable for their motor activities and spatial orientation perceptions in the new environment (64, 65). The completion of this fundamental adaptation can take weeks of constant exposure to novel situations, yet the temporal evolution of this adaptation is not instantaneous (66–68).

Astronauts who are newly exposed to microgravity may experience perceptual and functional deficiencies, such as errors in perception of spatial orientation and changes in locomotor and postural control (69). The capacity of crew members to carry out mission-critical operational duties, including controlling vehicles and using other complex systems, may be impacted by this sensorimotor impairment (70). Furthermore, it is believed that space motion sickness (SMS) is caused primarily by an adaptive central nervous system (CNS) that mistakenly interprets vestibular signals of self-motion as sensory information about self-orientation (71, 72).

The sensory-motor system consists of sensory and motor neurons. Of all systems within the human body, it is one of the most strongly affected by microgravity (73, 74). To adapt to microgravity, a reorganization of the central nervous system including the visual, vestibular, and somatosensory sub-systems must occur in order to process spatial information. When no gravitational fields are present, visual references are of utmost importance to determine one's orientation and position. An unaltered central vestibular system could send a signal that is misleading, leading to disorientation. Space adaptation syndrome (SAS) is caused by these conflicting signals from the visual and tactile senses, and inputs received by the vestibular organs, which leads to space motion sickness (SMS). The central nervous system has the potential to adapt to these conflicting signals. If the input received by the brain is transformed due to the change in gravitation, the central nervous system is obliged to develop new interpretations. If these new interpretations do not match with the patterns in our brains, SAS and consequently SMS could arise (47).

#### 3.1.4. Immune system and wound healing

The immune system is incredibly complex, comprising a plethora of different cell types, each with a specific role. While adaptive immune cells mount a delayed, antigen-specific response that results in long-term memory, innate immunity cells respond promptly and in a non-antigen-specific manner (75).

Immunity exhibits a notably translational nature, operating throughout the body, exchanging information, interacting with most systems, and being significantly affected by the likes of stress, diet, and exercise (76).

The immune system also reacts and is therefore sensitive to various types of stressors—psychological, physical, and local environmental (e.g., oxidative and radiation exposure). Negative effects on the immune system can occur when environmental extremes are prolonged or of high intensity, or disturb circadian cycles, alter diet or other factors that affect physiological and psychological stress (77, 78, 79).

Furthermore, immune system overactivity disorders that can lead to increased susceptibility to infectious diseases, such as allergy, asthma, eczema, and autoimmunity, have also been reported (80). As a result, the immune system is a special network of organs that is keenly sensitive to and responsive to changes occurring throughout the body (80).

Immune cells are also affected by microgravity, which could lead to immunodeficiency. The body would have great difficulties protecting itself from infections and tumours, as well as repairing itself. This decrease in performance of immune cells can stir up allergies and autoimmune pathologies, putting the mission at risk. Recent research has shown that during a 6-month orbital spaceflight, the human immune system is disregulated and latent herpes viruses can be reactivated (80).

Furthermore, during flight, some crew members report ongoing hypersensitive reactions. This phenomenon may elevate some crew clinical hazards during long space exploration missions when combined with galactic radiation exposure.

Part of this research field investigates T-lymphocytes in space. These cells are sub-divided into CD4+ T helper cells and CD8+ T cytotoxic cells (81). The T helper cells secrete cytokines to regulate the immune system's reaction to infections whilst the cytotoxic T cells induce cell death. A study on mice, performed in space, showed that the number of T cells diminished under microgravity conditions. The study indicated that spaceflights have a noticeable effect on the expression of cancer-related genes, and can therefore also increase carcinogenesis (81). This could result in astronauts developing cancer during long-duration missions. The development of carcinogenesis is increased further by the effects of microgravity on the natural killer cells (CD8+ T cytotoxic cells).

In conclusion, the frequent occurrence of immune abnormalities amongst crew members during low-earth orbit flights and the inability to predict which specific crew members will suffer such alterations provide clinical challenges for future missions to Mars.

Due to the impact of microgravity on immune cells and cytokines, the ability of the body to repair itself is also impaired in space. Furthermore, spaceflights negatively affect tissue repair and wound healing (82). The skin is the largest organ in the human body, comprising up to 16 percent of total body weight (83). Its purpose is to shield the body from environmental dangers, and, as a result, it serves as a barrier protecting the underlying tissues. Furthermore, the skin consists of various layers, each of which performs a specific role. During spaceflights, itches, rashes, and dry skin are the skin-related issues that astronauts most frequently report (84, 85). The International Space Station (ISS) environment, where the only options for skin hygiene are wet tissue wipes and non-rinse shampoos and soaps, is regarded to be a contributing factor to these problems. Atopic dermatitis, skin infections, and dryness and itching of the skin appear with increased incidence, in part because of other factors such as temperature, air circulation, and low humidity levels (86). Furthermore, changes in the skin's microbiota have been linked to higher skin infections and hypersensitivity in a cohort of nine astronauts, as well as a decrease in Proteobacteria (87). Finally, contact dermatitis, which is typically brought on by irritants like biosensors, tape, or electrode patches, is frequently reported. Delayed healing of wounds and minor cutaneous damage are also observed during spaceflight. On Earth, the wound healing process occurs in different stages. First, blood reaches the wound, together with platelets that form a fibrin clot. This clot prevents further bleeding at the wound site and ensures that wound healing can continue. However, in space, proteins and cells in the blood do not function normally, resulting in reduced wound healing efficiency (88).

One example is the creation of collagen, by fibroblasts, which is integral to the wound healing process. The production of this protein, however, is reduced in space, leading to a less efficient wound healing process. Due to the various effects of microgravity on the components of the wound healing process, the probability of successful wound healing is decreased. During long duration spaceflights or interplanetary missions, minor lacerations are unavoidable. Therefore, it is critical that further studies are performed to improve wound healing efficiency in microgravity (47).

### 3.2. Cellular effects

It is widely known that the mechanical cues cells receive from their surroundings influence cell behaviour (89, 90). Cells can detect changes in gravity, as well as changes in the mechanical properties of their surroundings, which stimulate biological reactions that can affect processes occurring at the cellular and tissue levels. Therefore, mechanical and gravitational forces can alter a number of processes, including gene expression, signalling, adhesion/migration properties, proliferation (91), differentiation (92) and apoptosis (93–95), as well as the way that cells organize themselves into three-dimensional (3-D) structures (constructs, tissues, and organs) (96).

Cells require gravitation for normal cellular processes. Therefore, in microgravity conditions, mechanical, biomechanical, and even physiological processes could be altered (46).

## 4. General risk assessments and mitigations

**Figure 5:**
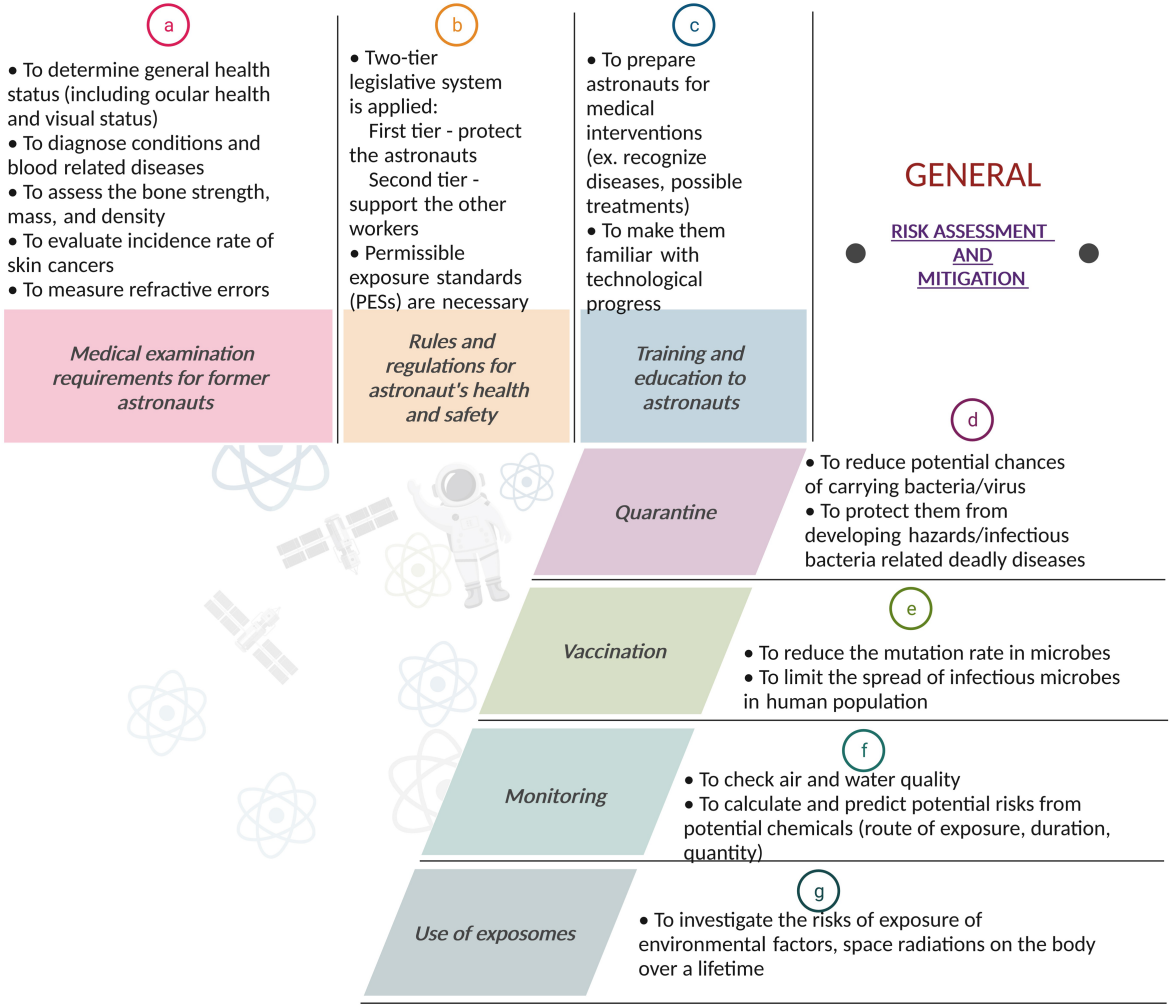
Risk assessment and mitigation: A flowchart-style depiction of the risk assessment and mitigation strategies for space missions. The figure outlines the systematic approach for identifying potential hazards, evaluating the risks of potential hazards, and implementing effective strategies to reduce or eliminate these risks. It highlights the importance of comprehensive risk management strategies to ensure mission success. Created with BioRender.com.

Figure 5 describes the general risk assessment and mitigation strategies used in space and are described below.

### 4.1. Medical examination requirements (MER) for astronauts

A range of medical tests is performed to ensure that astronauts are ready to go into space. These tests are conducted throughout the astronaut's working career, mostly on an annual basis. Most of these tests are conducted by all space agencies and are part of a long-term health follow-up programme for all astronauts to monitor potential occupational related injury or disease occurring during or after their space missions. These health programmes examine the incidence of various diseases, as well as acute and chronic morbidity and mortality of astronauts and define the risks associated with occupational exposures (80, 97–101).

By proactively monitoring and screening astronauts for occupational hazards, scientists can detect potential acute and chronic health problems at an earlier stage and possibly prevent progression of these health problems upon re-entry from space and over subsequent years.

A comprehensive review explaining all the various tests performed on astronauts upon their return from space can be found in (102). These include various blood, biochemical, musculoskeletal, dermatological, ophthalmological, audiological as well as cardio-pulmonary tests (102).

### 4.2. Rules and regulations according to health protection

To protect the health of astronauts, the various space agencies, such as NASA and ESA, estimate risks, set rules, and apply regulations, which may vary depending on the mission. A list of references that describes the risk methodology applied for astronaut protection in space can be found in (103–106).

These specific regulations must also be applied to touristic spaceflights. Furthermore, keeping these regulations up to date is essential as new hazards are continuously discovered, and their application could lead to planetary conflict if they were not relatively practical and universally applicable. The rules should be applied when there is no other alternative for reducing the exposure to a particular threat.

Another crucial element in the health legislation is the so-called permissible exposure standards (PESs) for radiation and for other risks (107). These standards are important for protecting astronauts from harmful exposures. If those standards were to be exceeded, health risks increase substantially, and heavy penalties or even imprisonment could be imposed. Unfortunately, these standards could also limit the work of astronauts. Hence, if the task is necessary for survival, exceptions can be made (108).

### 4.3. Training and education

Astronauts require medical intervention training to attend possible injuries that could occur during a mission (109, 110). This training gives the astronauts the necessary tools to recognize various diseases and possible treatments. Moreover, astronauts must be kept up to date with new scientific and technological developments in the form of regular training. As space exploration continues, new potential risks might be discovered (108).

### 4.4. Quarantine

Since the start of the Covid-19 pandemic, billions of people have been subjected to quarantine restrictions. However, astronauts have been familiar with quarantine since the dawn of the space age. There is a potential risk that astronauts may carry bacteria or viruses from earth into the spacecraft. To reduce this risk, astronauts are placed in quarantine for several days before the mission launch. Furthermore, pre-flight quarantine is not the only period where astronauts are quarantined. In space, the change in bacteria (more virulent) can increase the risks of contracting infections.

Space modules are small, sealed environments that are home to many different microbial ecosystems. Such contained communities are a substantial source of viruses, some of which can be harmful to humans and pose a risk to both personal and crew health (111, 112). Due to high radiation doses, microgravity, and compact spaces, space modules provide extraordinary conditions for Earth’s bacteria to proliferate and flourish (113).

If an astronaut does develop an illness, microbiologists and astronautical hygienists will need to characterize the hazard and act accordingly. This could be achieved by placing the infected crew member in quarantine. However, imposing a quarantine restriction in a spacecraft is not always possible due to its limited size.

### 4.5. Vaccination

Microorganisms, such as archaea that can be found in the extreme environments on Earth, are also present in space. These microorganisms are brought by the spacecraft into space and possess the ability to mutate. This can lead to astronauts contracting new diseases after exposure and potentially spreading them in the craft. Therefore, vaccination programs are implemented to limit the spread of infection and to protect individuals against these possible infections.

It is advised to implement a robust vaccination program that includes tetanus/diphtheria/acellular pertussis (Tdap), measles/mumps/rubella (MMR), influenza, pneumococcal, meningococcal, and hepatitis A and B vaccines (114). Varicella zoster virus vaccine should be administered due to the increased reactivation of herpes viruses, which has been observed in previous space missions (115).

### 4.6. Monitoring

Air- and water-quality must be monitored to ensure that no harmful substances are present during the mission. NASA developed specific tracers to follow up the presence of various pollutants such as carbon dioxide, because these substances could cluster in isolated spaces or create propellants or formaldehyde. Trace volatile organic analysers are used to monitor air quality. When potable water is recovered from urine, total organic carbon (TOC) analysers are used to ensure a satisfactory water quality. Many materials used in spacecraft expel different toxic chemicals. Toxicologists try to calculate and predict potential risks by analysing the route of exposure, duration of exposure, the amount of chemicals present during exposure—mixed and singular—as well as potential by-products of these chemicals. To follow up these different parameters, a variety of environmental monitoring sensors are present on the ISS (116). The monitoring of radiation is a special type of monitoring and will be described in the last section of this paper.

### 4.7. Biodosimetry

For more than 50 years, cytogenetic biodosimetry has helped to provide dose estimates for accidental radiation overexposures (117). Cytogenetics is the branch of genetics and cytology related to the study of chromosomes. Since the early 1990s, Cytogenetics has also been used on astronauts (118–120) by taking lymphocyte (a type of white blood cells) samples prior and after space mission to give an *in vivo* evaluation of radiation-induced damage induced during space travel (120, 121).

Biodosimetry provides a direct measurement of space radiation damage *in vivo*, taking into account the combined individual radiosensitivity of astronauts, the attenuation by the body, the shielding of the spacecraft and the combined influence of microgravity and other stress conditions on the body, all of which cannot be captured as accurately by physical dosimetry (122).

Biodosimetry of astronaut blood samples is incorporated into the astronaut medical records of European and Canadian astronauts and is a medical requirement as specified in the International Space Station (ISS) Medical Evaluation Document (123, 124). Maintaining reliable, standardized biodosimetry techniques are crucial as longer-duration space travel is being considered.

### 4.8. Use of exposomes

The exposome is the collection of all exposures to environmental factors on our body from inception to death (125). Exposure to environmental factors can be exogenous or endogenous in nature, including diet, chemical exposure, stress, and use of alcohol. Exposome science is interested in non-genetic explanations to disease development. Therefore, the use of exposome monitoring could aid in ascertaining whether particular astronauts are more prone to developing diseases after exposure to certain space-related risks such as radiation. Astronauts could be screened on both terrestrial and extra-terrestrial factors to identify exposomes related to certain exposures. This could allow astronauts who are less likely to develop diseases following the exposure to specific hazards to be selected for missions that encounter such hazards. However, the ethics of restricting certain astronauts from participating in missions makes the use of exposomes not as straightforward as it might seem. European legislation does not permit ESA to restrict an astronaut based on genetic analysis. Doing so is akin to discrimination, which the European Union is against. Who will make the decision to restrict an astronaut from going into space?

Will the insurance fee for an astronaut more prone to developing illnesses be higher? Will the astronaut face restrictions and penalties during the mission, such as limited Extravehicular activities due to his/her higher risk susceptibility? If, after several studies, the use of exposomes indicates that certain races or sexes are more prone to developing diseases after exposure to specific hazards, is it ethically correct to restrict this population of astronauts from going on certain missions? Who can get access to this data? The use of exposomes seems at first a promising avenue for investigation, but once the questions about how the data will be used arise, it becomes complicated to justify (108).

## 5. Radiation risk assessments and mitigations

### 5.1. Countermeasures for space radiation

Since radiation risk is directly related to dose, a mitigation strategy must be developed to reduce the dose absorbed to an acceptable level. The radiation dose depends on the mission scenario, i.e., the type and intensity of radiation to which the astronauts are exposed, as well as the duration of the mission, the spacecraft's attitude, and solar activity. Therefore, these different mission parameters can have a drastic impact on the radiation dose absorbed by astronauts. For example, the average dose received by astronauts inside the ISS is approximately 0.5–1 mSv/day (126). For a mission to Mars, the radiation dose will differ from those on board the ISS. In deep space, the Radiation Assessment Detector (RAD) device [on board the unmanned Mars Science Laboratory (MSL) “Curiosity”] measured a mean GCR dose-equivalent rate of 1.84 mSv/day (13), with an estimated dose equivalent for the Martian surface of 0.64 mSv/day (127).

**Figure 6:**
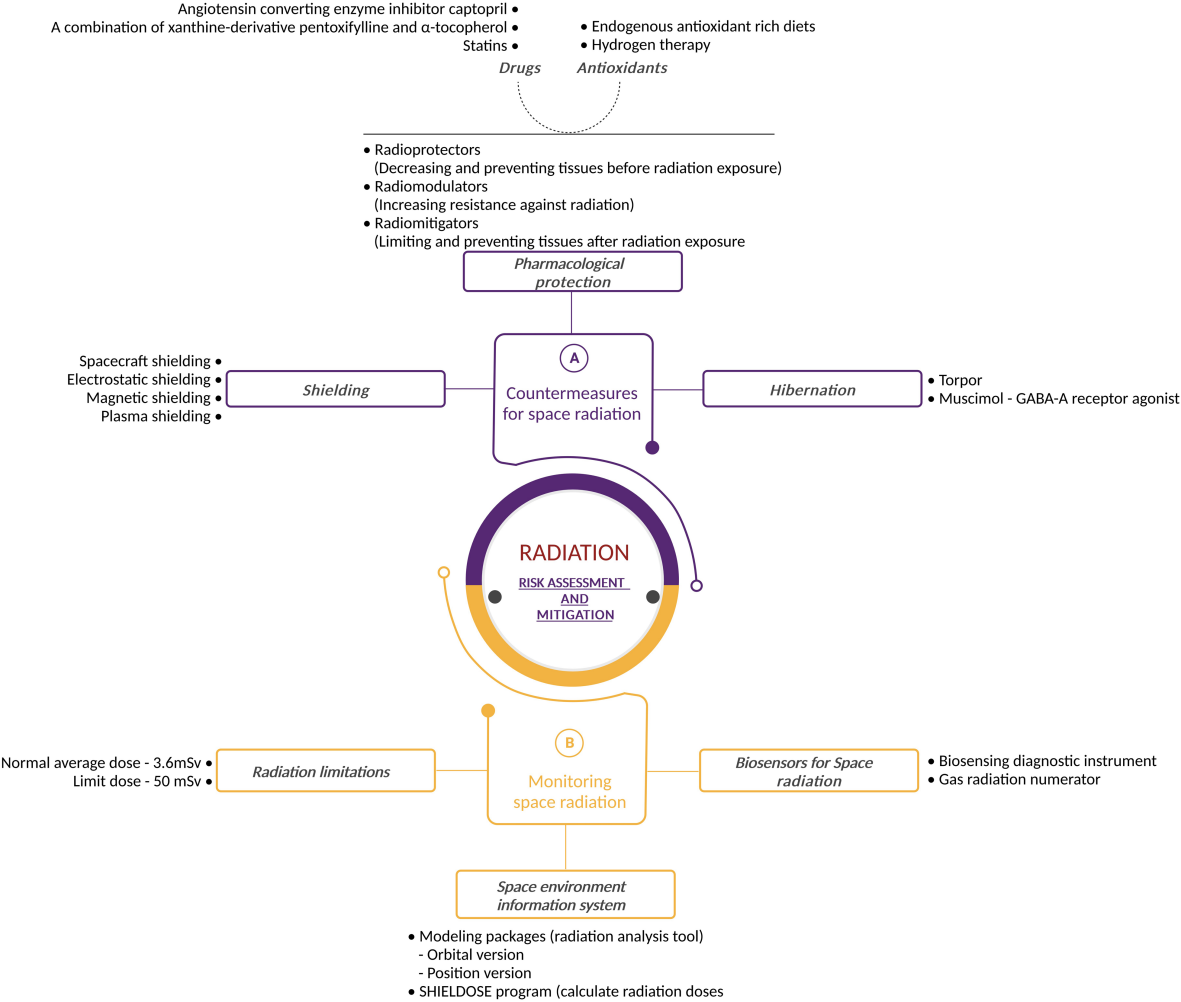
Radiation risk assessment and mitigation: detailed illustration outlining the radiation risk assessment and mitigation strategies used during space exploration. The figure highlights countermeasures such as shielding and safe exposure duration, pharmacological protection and hibernation, emphasizing the multidisciplinary approach to managing radiation hazards. It also portrays space radiation exposure assessment and monitoring strategies, space environment information system as well as biosensors for space radiation. Created with BioRender.com.

Two types of countermeasures exist to protect astronauts from space radiation, the first being to reduce the radiation exposure to an as low as reasonably practical (ALARP) dose by using space shielding (section 5.1.1). The second involves biological countermeasures through the use of radioprotectors and radiomitigators, genetic approaches and hibernation (section 5.1.2). Figure 6 describes the various possible risk assessment and mitigation strategies.

#### 5.1.1. Shielding

Shielding is a logical approach to enduring the brunt of space radiation. Unfortunately, it is more complex than it may appear.

Nowadays, the majority of spacecraft are sufficiently protected from space radiation within LEO. However, outside LEO, these forms of shielding will not provide an adequate barrier to protect astronauts. Protection against SPE particles is possible with spacecraft shielding, but the heavy elements produced by GCR ions have such large amounts of energy that it is nearly impossible to prevent them from entering the vehicle (128).

Physical based mitigation methods aim to reduce the radiation dose by acting on the incoming radiation field in two different ways: (i) active shielding, in which electromagnetic fields deflect or trap cosmic rays, and (ii) passive shielding, which exploits nuclear and electromagnetic interactions between incident radiation and materials (129).

Studies have shown that active shielding could be the solution, several types of active shielding have been proposed. Electrostatic shielding operates by creating an electric field around the spacecraft to slow down negatively charged particles (130–135). However, the effects of living in a giant electrostatic field are not fully known. Therefore, studies investigating this technology must be performed. Another type of active shielding is magnetic shielding (136–139), in which solenoids generate a magnetic field around the spacecraft to either deflect or trap charged particles. Lastly, plasma-shielding uses ionized particles to form a magnetic field around the spacecraft (140–145). These particles could entrap or deflect charged radiation particles. This protection works threefold since it combines both electrostatic and magnetic shielding, together with the ionized particles. Advantages and disadvantages of these methods have been discussed in (146–148). Although active shielding remains an area of ongoing research, it is not clear whether an active shielding solution can provide adequate protection against the high-energy GCR components of the space radiation field (149) whilst still being practically feasible for space travel.

Significant research has been performed in the field of passive shielding to optimize material selection and shielding in the various space radiation environment scenario (150–153).

Previous studies have shown that conventional shielding methods (such as polyethylene or aluminium) of specific thickness cannot provide sufficient capacity of shielding against the ion component HZE, which is the biggest threat to astronauts when embarking on long-term voyages (154, 155).

The increased shielding thickness beyond standard spacecraft values (average thickness of about 10 g cm^−2^) is required to reduce the risk of radiation below the Space Permitted Exposure Limit (SPEL) (156). However, with the current NASA launch capabilities, the increased shielding exceeds mass limits for launches set by NASA (16, 157). This limit refers to a predetermined threshold of mass established by NASA and indicates the maximum allowable mass that must not be exceeded during the launch. It is a critical criterion that ensures the safety and well-being of astronauts and spacecraft equipment throughout their journey. A solution is therefore needed to reduce radiation exposure below regulatory limits, without compromising launch restrictions. For this reason, the current approach is to reduce the dose by modifying the radiation field composition via nuclear fragmentation, namely by breaking ions into particles with lower charges and similar velocities, while avoiding the dose increase of the mixed field induced by the secondary particles (158).

Passive shielding methods could provide sufficient shielding on the lunar and Martian surfaces. This could be achieved by building the surface habitats from regolith (159, 160). Another shielding approach to reduce radiation exposure during a stay on the surface relies on analysing the lunar geography to take advantage of particular geographical features when selecting the construction site.

Despite these major discoveries, further studies are needed to implement them in current and future spacecraft designs (16). ESA has been supporting theoretical and experimental studies on space radiation shielding (ROSSINI) (152), aiming at dose reduction through improvement of structure configuration and development of new materials. Lithium hydride (LiH) compounds were identified as possible alternatives to polyethylene (currently used on the ISS) for radiation shielding (161).

Boron nitride nanotubes (BNNTs) spun into yarn, making it very flexible, have also been studied (162). Research has been performed by sampling regolith and studying the lunar geography to identify suitable materials for radiation protection (163). Results showed that lava tubes and lunar regolith could be used as shielding materials against radiation particles, as well as for controlling the thermal environment (164, 165).

Finally, hybrid active-passive shielding approaches, where active shielding (comprising an electrostatic shielding configuration consisting of multiple charged planes and charged rods) was placed outside passive shielding have been studied to investigate the efficacy of combined passive and active shielding for protection against extreme solar particle events (SPEs) and free-space GCR spectra under solar minimum and solar maximum conditions (166).

#### 5.1.2. Biological protection

In parallel to shielding, scientists are also considering alternative methods of radiation protection. Pharmacological protection could ensure that radiation does not damage the astronauts’ bodies or that any such damage is repaired with a high degree of fidelity. McLaughlin et al. divided this form of protection into three categories: radioprotectors (preventing the harmful effects of radiation exposure on biological tissue); radio modulators (increasing the body's resistance to radiation by giving a substance prior to actual radiation exposure) and radiomitigators (limiting and preventing tissue degeneration after radiation exposure). Drugs and antioxidants could ensure that the pharmacological protections described above would work in the human body (167).

##### 5.1.2.1. Radioprotectors and radiomitigators for spaceflight

Radioprotectors and radiomitigators are potentially interesting compounds for future deep space missions (167). Radioprotectors and radiomitigators increase the resistance of the body to radiation exposure by increasing the effectiveness of antioxidative protection and by neutralising radiation effects (radioprotectors) or by accelerating the restoration of tissue following exposure (radiomitigators) (168). Radioprotectors are molecules that protect tissues from direct and indirect damage induced by ionizing radiation whilst radiomitigators can reduce radiation-induced damage or promote repair, depending on whether they are administered pre or post radiation exposure (168).

Most radioprotectors (and radiomitigators) are antioxidants, and thus act as free-radical scavengers. They can therefore increase the levels of antioxidant defences, such as reduced glutathione and/or of antioxidant enzymes such as superoxide dismutase, glutathione peroxidase and thioreductase. They can also stimulate one or more DNA repair pathways, prevent apoptotic cell death, modulate redox sensitive genes, and display an anti- inflammatory response as well as promoting tissue regeneration (168).

For radioprotectors to be suitable for spaceflight usage, they should reduce or prevent direct and indirect effects of ionizing radiation on cells and healthy tissues in animals and humans (whilst not protecting tumour cells). Furthermore, they should not be toxic, nor cause any adverse effects when administered alone or in combination with other drugs. They should have a sufficiently broad window of effectiveness once administered, have a long and stable shelf life, and be easily accessible without complex handling or transport arrangements. In recent decades, various natural as well as synthetic compounds have been brought forward and investigated for their potential as radioprotectors (169).

There exists a variety of currently approved agents that show preclinical and clinical efficacy in preventing or reducing acute and chronic sequelae of radiation exposure (167). Many of these agents could be combined with vitamins, nutraceuticals, or other endogenous substances to obtain synergistic effects (170, 171).

Amifostine, for example, as well as other thiol compounds and their derivatives have already been used clinically on cancer patients on Earth to prevent complications from radiotherapy treatment. However, the practical applications of most of these synthetic compounds remain restricted due to their limited administration routes, their narrow administration window for efficacy and their toxicity at high doses or during recurrent usage, making them far from ideal candidates for use by astronauts (172).

To conclude, antioxidants appear to offer benefits to human health in space. Although the future for antioxidants seems promising, insufficient data are available to reach a positive conclusion about their use in space.

##### 5.1.2.2. Gene therapy for deep space exploration

Other strategies to increase human radiation resistance to withstand long exposures to cosmic radiation are being investigated (173). The first such approach entails inducing an overexpression of endogenous and exogenous antioxidants via integration and expression of their cognate transgenes (174). Various studies using this approach have reported a substantial reduction in early and late-stage irradiation damage following the delivery of magnesium superoxide dismutase (MnSOD) transgenes systemically or in a tissue-specific manner by using plasmid/liposomal vectors (175, 176).

A second strategy is to induce an overexpression of endogenous and exogenous DNA repair genes (such as overexpression of O6-alkylguanine DNA alkyltransferase and yeast AP endonuclease) to enhance the efficiency of endogenous DNA repair and to reduce mutagenesis and associated carcinogenesis (177, 178).

A third option aims to increase the expression of endogenous and exogenous radioprotective transgenes, such as the promising nuclear protein Damage suppressor, (Dsup), discovered in the radiation resistant tardigrades (173, 179, 180).

The fourth strategy consists of characterising the genetic determinants of high human radioresistance in conjunction with clinical translation via multiplex genetic engineering. For example, single-nucleotide polymorphisms (SNPs) are genomic variants at a single base position in the DNA. SNPs occur at specific positions within the genome and are present in at least 1% of the human population. Part of the individual differences in radiosensitivity within the human population can be attributed to differences in SNP profiles. The set of candidate SNPs that yield high radioresistance could be identified, making the use of targeted gene editing technologies (e.g., CRISPR/Cas9) to replicate those SNP variants in individual humans possible (181, 182).

Finally, an additional option is the generation and characterisation of enhanced radioresistance via experimental evolution in conjunction with clinical translation via multiplex genetic engineering (183). This strategy would take advantage of experimental evolution to generate populations of model organisms or human cells with enhanced radioresistance through exposure to high levels of radiation. The surviving cells or organisms are then bred, followed by the characterisation of the genetic determinants of the resulting enhanced radioresistance phenotype and the subsequent clinical translation of such genetic determinants to humans via multiplex genetic engineering. This technology was successfully implemented in the bacteria E. coli (184).

##### 5.1.2.3. Hibernation

A more recent approach to radiation mitigation is found in the form of hibernation. This state can be observed in mammals as a means of surviving periods of reduced resource availability or adverse meteorological conditions. Hibernation has long been known to reduce the impact of radiation damage, in addition to its protection against famine and starvation periods (185). Hibernation induces a remarkable resistance to radiation damage, which could help mitigate the effects of radiation exposure experienced during spaceflights (186, 187).

Torpor is an animal's metabolic condition during hibernation. During torpor, the metabolism is drastically reduced over a period of a few hours to several weeks. Scientists are investigating whether the state of torpor could be induced during long manned space trips to Mars and beyond (188). Natural torpor's decreased metabolic rate is associated with deep biochemical changes, such as the switch from glycolysis to lipolysis and ketone consumption, extensive but reversible changes in organs including the brain and kidney, and Ca^2+^-mediated heart rate control (188).

The lowering of astronauts' physiological needs through intermittent or constant torpor states (that mimic those experienced naturally during hibernation) could potentially be groundbreaking for deep space missions (189). If astronauts could endure this physiologically natural state, it would significantly reduce the number of supplies and the size of the habitat needed, but would require constant monitoring and artificial intelligence (AI) assisted monitoring of the crew (189). It would also mitigate radiation and low-gravity side effects, potentially reducing neurobehavioural effects such as boredom, loneliness, and aggression. Furthermore, up to 75% less water and food could be consumed, which would reduce both the spacecraft's cargo requirements and excreta production (189).

Recently, the comprehensive Mission Concept and Requirements Assessment (MicRA) study was carried out by the European Space Agency (ESA) to evaluate the potential and the research needs for the use of torpor as a tool for human spaceflight (189).

All hibernators, studied so far, have shown increased radiation resistance when in their torpid state (185). Recent studies have examined the fundamental mechanisms and their applicability to space missions (186, 190). Therefore, under prolonged physical stress, starvation, low metabolic rate, and low body temperature, torpor and hibernation have been shown to offer a robust state of tolerance and tissue preservation, as well as radiation resistance.

Scientists are currently developing artificial methods of torpor, where GABA-A receptor agonist—“muscimol” is injected into the brainstem region. This induced torpor would increase radioprotection (191). Currently, research has been performed successfully on zebra fish (192) and rats. Torpor decreases cell death (apoptosis) (193) by up-regulation of anti-apoptotic proteins such as Bcl-XL in an organ-specific manner (194, 195), and the necrotic tissue injury seems to be minimal (196).

However, more studies are needed to appreciate the complexity of the radiation resistance mechanism before applying it fully to astronauts (188).

### 5.2. Monitoring space radiation

#### 5.2.1. Radiation limitations for astronauts

During all stages of flight and even post flight, astronauts are affected by space radiation which can be considered an imperceptible risk (197).

In addition, the nature of space radiation is unique compared to terrestrial radiation, and data on the effects of space radiation on humans are extremely sparse. Exposure data are only available from 24 astronauts on 12-day missions outside LEO (3). This lack of data requires space radiation risk models to rely on terrestrial radiation exposure data, which may have different physiological effects than those encountered in space (16, 198).

Furthermore, a key duty for medical practitioners is to understand, foresee, and minimize potential health risks during space flight (199). To that end, guidelines have been put in place for radiation doses that humans can absorb during a given period.

The amount of radiation absorbed by human tissues is measured using the unit Sievert (Sv) (200). The yearly average dose equivalent for humans is 3.6 mSv resulting from natural exposure on Earth. However, this is a relatively small dose compared to the doses encountered when working in a radiation rich environment. The radiation protection regulations from the International Commission for Radiological Protection (ICRP) set the limit for occupational exposure to 20 mSv per year of effective dose, averaged over a defined period of 5 years (100 mSv in 5 year), with no yearly dose exceeding 50 mSv (200). However, astronauts are exposed to significantly higher amounts of radiation than the average worker on Earth. Furthermore, astronauts encounter different types of radiation when in space. Therefore, these guidelines are not sufficient to protect astronauts for all types of space travel. To maintain crew doses below set limits, or as low as reasonably achievable, the ionizing radiation environment must be monitored throughout a space flight, meaning crewmembers' critical organ and tissue doses are monitored and assessed. The data collected are recorded for each mission, as well as for cumulative career records (124). This enables immediate countermeasures to be implemented in the event of transient radiation exposure events (such as during EVA: solar particle events or electron belt enhancements).

To keep astronauts safe but delve deeper into space, the ICRP has defined three fundamental guiding concepts that should be adhered to during human spaceflight. These concepts complement the ICRP's effective dose regulation, mentioned previously. The first of these three guiding concepts is justification. Report 60 of the International Commission on Radiological Protection (ICRP) states that “*no practice involving exposures to radiation should be adopted unless it produces sufficient benefit to the exposed individual or to society to offset the detriment it causes*” (201).

The second principle, Optimization, is defined as “*…a process or method used to make a system of protection as effective as possible within the given criteria and constraints*”. Thirdly, the principle of Limitation states: “*individual dose limits are applied to ensure that the principles of justification and ALARA (as low as reasonably achievable) are not applied in a manner that would result in individuals or groups of individuals exceeding levels of acceptable risks*” (201).

The effective dose regulation and the three guiding concepts provide a basic framework to manage radiation exposures related to human spaceflight. However, to fully comprehend and communicate detailed information about potential radiation related risks to a specific astronaut or to a space mission, more metrics and uncertainty evaluations are needed. Future missions will travel to distant locations, well beyond LEO. These will present new design and radiation protection challenges (202). A list of current operational radiation limitations utilized by the ISS partners can be found in (199) and updates can be found in (203, 204).

An astronaut's annual and mission-related medical examinations, medical certification, and re-certification procedures all address radiation in space and its associated possible health risks (199). During these extensive medical examinations, the general organ functions, the blood-forming organs, the central nervous system, and the endocrine system in particular, are observed, analysed and assessed. In addition, pathogenetic factors, as well as potential new risk factors, are monitored (199). For an astronaut's mission assignment, pre-flight crew radiation exposure histories are reviewed and analysed.

Recently, NASA has modified the previous risk-based radiation limits for long-term deep space flights to a new limit of 600 mSv total effective dose over an astronaut's career (205), based on the recommendation of the National Academies of Sciences, Engineering, and Medicine (206). Previously, a risk-based limit was imposed by NASA that ensured a career-integrated 3% risk of exposure-induced death (REID) at the upper 95% confidence interval (156). The new radiation limit removes the dependence on astronaut age and biological sex, making future space travel more inclusive and removing the inherent complexity in computing REID. In addition to the total effective dose for a career, short-term limits exist to restrict doses to prevent specific tissue reactions (also known as non-carcinogenic or deterministic effects) to organs, such as skin, lens, blood forming organs and the circulatory and central nervous systems (205).

#### 5.2.2. Modeling the space environment

To ensure that astronauts and electronics can tolerate the radiation environment found in space, even whilst being shielded by the spacecraft/habitat, the radiation environment must first be determined during the mission planning and design stages. This requires instrumentation that can provide the necessary data, as well as analysis of existing data from current detection systems, such as those on the lunar surface (207) or in other locations (208).

Accurate space radiation models are crucial for lowering the risks astronauts encounter and for building high-performance, low-cost space equipment. The AP-8 (209) and AE-8 (210) models of the Earth's radiation belts are commonly used models.

The AP-8 models of trapped protons, which are appropriate for solar maximum and minimum, respectively, include AP-8 MAX and AP-8 MIN. Both AE-8 MAX and AE-8 MIN are included in the AE-8 models for trapped electrons. These common models are praised for their thorough spatial coverage and user-friendliness, although they have drawbacks and suffer from some inaccuracies (211–214).

However, both models, AP-8, and AE-8, were constructed using data that were gathered between 1958 and 1979. The models might no longer accurately represent the environment that today's space systems experience due to the dynamic nature of the space environment. Notably, it is known that the Earth's inner radiation belt is tainted by the high-altitude nuclear device detonations that occurred in the late 1950s and early 1960s (211).

New standard radiation belt environment models are thus required because modern applications require more precision, functionality, and energy coverage that AP-8 and AE-8 do not deliver. Therefore, newer systems were implemented. For example, the SPENVIS is an ESA's space environment information system used to simulate and model the space environment (https://www.spenvis.oma.be/). It includes models used for the analysis and prediction of radiation environments from galactic cosmic rays, solar energetic particles, natural radiation belts, plasmas, gases, meteoroids, and debris. SPENVIS uses the Jensen and Cain 1960 internal field model for AE-8 MIN and MAX, as recommended in (212). It also models the space impact on the spacecraft faces and systems during flight into orbit by using the Jenson and Cain model (212).

Thereafter, the computer code SHIELDOSE program (215) can help to calculate the radiation doses based on the shielding, the damage to solar cells, LET spectra of cosmic rays and energetic particles and make estimations of single-event disturbances for micro-electronics and equipment.

Other publicly available models can also be found. For proton models, PSB97 (216), Low Altitude Trapped Radiation Model (LATRM) (217), Trapped Proton Model (TPM-1) (218), and the Combined Release and Radiation Effects Satellite Proton model (CRRESPRO) (219) have been developed since AP-8.

Electron models that were developed since AE-8 include the Combined Release and Radiation Effects Satellite Electron model (CRRESELE) (220), Flux Model for Internal Charging (FLUMIC) (221), and the Particle ONERA2-LANL3 Electron (POLE) model (222).

#### 5.2.3. Detectors and biosensors for space radiation

Each astronaut on board the ISS has their own passive dosimeter. The radiation levels are monitored regularly by active and passive detectors throughout the entire station. A gas radiation numerator, called Tissue Equivalent Proportional Counter (TEPC), estimates the radiation dose absorbed by crew members. Furthermore, the detector is designed to function like a human body. The detector has a plastic casing that mimics human tissue whilst the internal gas simulates human cells.

However, to obtain more reliable radiation measurements linked to biological response, various biosensors are being developed. One example of research on radiation is the nanosatellite BioSentinel, launched from Artemis-I, that will perform *in situ* biological tests on living organisms (yeast) in the radiation environment of deep space (223). On-board active dosimetry will take measurements of the radiation field found within the spacecraft after having passed the shielding and internal components found within the BioSentinel nanosatellite (224). The primary objective will be to assess the damage and reaction in cells exposed to radiation during spaceflight. This enables scientists to measure the effects of space radiation on living organisms over long periods of time beyond LEO (224, 225). In view of future deep space missions, other biosensor devices are being tested within the Artemis programme (226, 227).

## 6. Conclusion

In this review, the investigation of ionizing radiation's causes, effects, and health hazards highlights the importance of effective radiation safety regulations and mitigation strategies for use during extended deep space missions. To achieve safety standards for future deep space missions, innovative shielding techniques will be required because solar particle events and galactic cosmic radiation pose a serious health risk to astronauts. Furthermore, the necessity for specialized medicinal countermeasures reviewed in the biological section of this review is further highlighted by the wide range of adverse health impacts, such as tissue degradation, carcinogenesis, and central nervous system damage. The thorough analysis of the effects of microgravity on many physiological systems reveals how crucial it is to devise mitigation strategies to counteract these effects. The in-depth analysis of risk assessment techniques, including the rules, and guidelines for medical examinations, emphasizes how healthcare and safety protocols must continuously change to maintain astronaut safety. In order to reduce health risks during space missions, quarantine, immunization, and monitoring become essential processes as discussed in this review. Exposomes and biodosimetry illustrate the multidisciplinary approach necessary to thoroughly assess astronaut health. Additionally, research into radiation protection techniques including shielding, biological protection, radioprotectors, as well as cutting-edge methods such as gene therapy and hibernation offer additional solutions to radiation related health hazards. The use of these techniques, as highlighted in this review, could have significant potential for facilitating deep space exploration whilst ensuring astronaut safety.

In conclusion, the complex interactions between ionizing radiation, microgravity, and astronaut health during space travel are highlighted in this review. The urgency to establish effective risk assessment and mitigation strategies is driven by the impact these stressors have on different physiological systems. This review highlights potential avenues that could be stepping stones in astronaut safety for upcoming space exploration missions. It is important to mention that although methods to protect the health of astronauts already exist, innovative technologies are being studied and developed to push the boundaries of where Humans can travel to. However, there is still a need for extensive research in this field. As we continue to collect data on the vital signs of astronauts' bodies, we can broaden our understanding of the risks encountered during space exploration. Moreover, we must continue to produce new risk assessment methods appropriate for both current and future risks.
